# Production of Siderophores by an Apple Root-Associated *Streptomyces ciscaucasicus* Strain GS2 Using Chemical and Biological OSMAC Approaches

**DOI:** 10.3390/molecules26123517

**Published:** 2021-06-09

**Authors:** Reyhaneh Armin, Sebastian Zühlke, Gisela Grunewaldt-Stöcker, Felix Mahnkopp-Dirks, Souvik Kusari

**Affiliations:** 1 Center for Mass Spectrometry (CMS), Faculty of Chemistry and Chemical Biology, Technische Universität Dortmund, Otto-Hahn-Str. 6, 44227 Dortmund, Germany; reyhaneh.armin@currenta.biz (R.A.); sebastian.zuehlke@tu-dortmund.de (S.Z.); 2 Institute of Horticultural Production Systems, Section Phytomedicine, Leibniz Universität Hannover, Herrenhäuser Str. 2, 30419 Hannover, Germany; grunewaldt@ipp.uni-hannover.de; 3 Institute of Horticultural Production Systems, Section Woody Plant and Propagation Physiology, Leibniz Universität Hannover, Herrenhäuser Str. 2, 30419 Hannover, Germany; mahnkopp@baum.uni-hannover.de

**Keywords:** Apple Replant Disease (ARD), HPLC-HRMS, HPLC-HRMS/MS, One Strain Many Compounds (OSMAC), siderophores, *Streptomyces ciscaucasicus*

## Abstract

Apple Replant Disease (ARD) is a significant problem in apple orchards that causes root tissue damage, stunted plant growth, and decline in fruit quality, size, and overall yield. Dysbiosis of apple root-associated microbiome and selective richness of *Streptomyces* species in the rhizosphere typically concurs root impairment associated with ARD. However, possible roles of *Streptomyces* secondary metabolites within these observations remain unstudied. Therefore, we employed the One Strain Many Compounds (OSMAC) approach coupled to high-performance liquid chromatography-high-resolution tandem mass spectrometry (HPLC-HRMS^n^) to evaluate the chemical ecology of an apple root-associated *Streptomyces* *ciscaucasicus* strain GS2, temporally over 14 days. The chemical OSMAC approach comprised cultivation media alterations using six different media compositions, which led to the biosynthesis of the iron-chelated siderophores, ferrioxamines. The biological OSMAC approach was concomitantly applied by dual-culture cultivation for microorganismal interactions with an endophytic *Streptomyces pulveraceus* strain ES16 and the pathogen *Cylindrocarpon olidum*. This led to the modulation of ferrioxamines produced and further triggered biosynthesis of the unchelated siderophores, desferrioxamines. The structures of the compounds were elucidated using HRMS^n^ and by comparison with the literature. We evaluated the dynamics of siderophore production under the combined influence of chemical and biological OSMAC triggers, temporally over 3, 7, and 14 days, to discern the strain’s siderophore-mediated chemical ecology. We discuss our results based on the plausible chemical implications of *S. ciscaucasicus* strain GS2 in the rhizosphere.

## 1. Introduction

The recultivation of plants in the soil where identical species were previously cultivated may reduce plant growth and lower fruit yield and quality [1]. This problem can affect trees, such as apples, pears, cherries, and roses. Apple Replant Disease (ARD) is a frequently occurring problem in replanted apple orchards. Over the past few decades, a continual decrease in areas of apple plantation was observed. Not only across Europe and the USA, but also across the globe, for example, the Bohai Gulf, one of China’s main apple production areas, was affected by this disorder [2,3]. It is, thus, of vital interest to find the underlying etiology of this problem. Interestingly, not all apple tree genotypes show the same ARD symptoms; some are vulnerable, while others are somewhat tolerant [4,5,6]. The damage of ARD extends from belowground roots to aboveground tissues such as leaves and stems. On the one hand, roots display signs of browning and blackening, tip necrosis, and reduced root hairs [5,7,8,9]. On the other hand, above the soil, stunted plant growth, and reduced plant biomass are typically observed [10,11,12]. There is a decrease in the fruit yield and size, along with the deterioration of taste. Deficiency in soil nutrients, dysbiosis of the soil microbiome and apple root endophytic microbial community, and residual herbicide activity encompass some of the typical elements contributing to ARD [3,13].

However, recent investigations suggested that the disease’s main reason may lie in biological factors such as nematodes, the lack of beneficial bacterial groups, and pathogenic fungi. The latest research has defined ARD’s primary etiology as a complex of multiple pathogenic fungal species that act synergistically, causing apple plants’ improper growth [5,7]. Mazzola and Manici (2012) proposed several candidate pathogenic agents responsible for ARD, including *Fusarium* spp., *Pythium* spp., and *Cylindrocarpon* spp., presently updated as members of the family Nectriaceae with diverse genera [14,15,16,17,18]. Different Nectriaceae genera, species, and anamorphs (formerly *Cylindrocarpon*), such as *Ilyonectria* and *Dactylonectria* are associated with ARD [19,20,21]. Nectriaceae (*Cylindrocarpon*) species are pathogens known to colonize the roots of trees in a forest ecosystem. They are mainly associated with the root decay and bark necrosis of their wide host range. Four species, namely *C. destructans, C. liriodendra*, *C. macrodidymum,* and *C. pauciseptatum* have been linked to ARD. For example, Tewoldemedhin et al. (2011) reported that these species, specifically *C. destructans*, caused lesion development on various seedling roots. *C. destructans*, which was later classified as a new genus called *Ilyonectria* [22], may either cause a rotting root or trigger a “rusty” root in plants [23]. While the rotting root is a result of a complete invasion of plants by the pathogen through the excretion of fungal enzymes, the “rusty” root is a defense mechanism employed by plants by producing iron phenolic compounds. Other species, such as *C. olidum*, which are not as aggressive, are known to inhibit the growth of other microorganisms by producing active components such as cannabiorchichromenic acid [24]. The growth of these fungi is also dependent on factors such as soil pH and the age of roots [25]. Besides, high quantities of minerals such as iron in the environment enhance their growth and successful attack on the roots [25]. Therefore, it may be a crucial challenge for the host plant and its associated microbiome to contribute to the defense system by taking up the ferric ions in the environment or releasing secondary metabolites that intoxicate such pathogens.

Recent molecular investigations have unraveled a striking phenomenon of selectively increased abundance of actinobacteria, typically *Streptomyces* species, in roots grown in ARD soils compared to healthy roots grown in non-ARD soils [26]. A negative correlation between increased shoot length and fresh biomass and an increased abundance of *Streptomyces* in apple roots grown in ARD soils was also noted. In another study, selective enrichment of *Streptomyces* species in the rhizosphere in ARD soils in split-root experiments was demonstrated by gene sequencing [27]. Noteworthy, damage to apple plant roots associated with ARD typically coexists with endophytic and rhizosphere microbiome dysbiosis and discriminatory amelioration of *Streptomyces* species [9,26,27,28]. However, the causality or correlatedness of these observations and the impact of *Streptomyces* species on ARD etiology remains unexplored [26,28]. Remarkably, the chemical-ecological role of secondary metabolites or specialized natural products produced by *Streptomyces* species with niche-specific biological activities facilitating the molecular and biological observations is mostly unknown.

**Figure 1:**
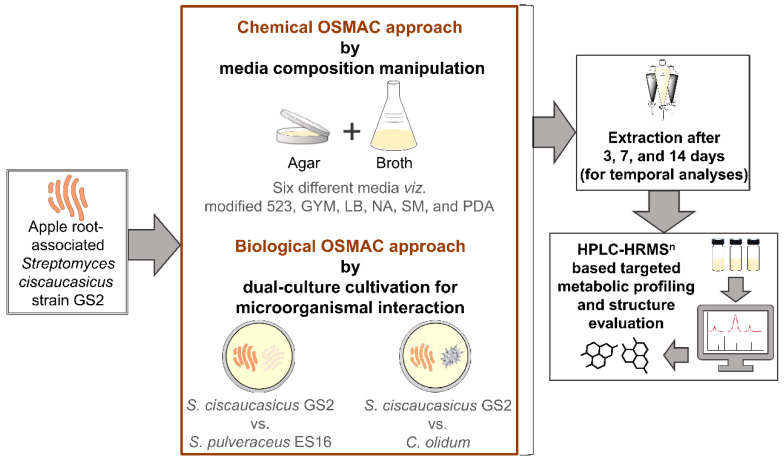
Schematic representation of the overview of workflow employed in the present study. GYM, glucose yeast malt medium; LB, lysogeny medium; NA, nutrient agar; SM, streptomyces medium; PDA, potato dextrose agar.

Against this background, we investigated a cultivable apple (*Malus domestica*) fine root-associated *Streptomyces ciscaucasicus* strain GS2 that we isolated within the Central Experiment 2 setup (CE2) of the BMBF *BonaRes* program *ORDIAmur* [9]. In the present study, we report the production and modulation of siderophores (compounds **1**–**8**) by *S. ciscaucasicus* GS2 under the influence of chemical and biological One Strain Many Compounds (OSMAC) approaches [29,30,31,32]. We applied the chemical OSMAC approach encompassing media composition manipulation by cultivating the strain in six different media compositions ranging from nutrient-rich to minimal conditions, including media to stress the organism under submerged conditions (broth) as well as on agar (Figure 1). Under the influence of different chemical OSMAC conditions, we investigated and characterized the siderophores produced by the strain over 3, 7, and 14 days (temporal evaluation), using high-performance liquid chromatography-high resolution tandem mass spectrometry (HPLC-HRMS^n^). After that, we employed the biological OSMAC approach by dual-culture cultivation to study the siderophores’ production and modulation triggered by microorganismal interactions (Figure 1). On the one hand, to explore how *S. ciscaucasicus* GS2 might mediate mutualistic interaction in soil with other species of the same genus using siderophores (e.g., using similar siderophore receptors), we co-cultivated it with *S. pulveraceus* strain ES16 that was isolated as an endophyte from apple roots. On the other hand, we co-cultivated it with the phytopathogen *Cylindrocarpon olidum* (DSM 62520) to explore the implications of triggered siderophores during the strain’s interaction with a different genus it might encounter in soil. The dual-culture experiments were performed in all six chemical OSMAC media conditions and temporally over 3, 7, and 14 days, coupled to HPLC-HRMS^n^ analyses, to discern the siderophores’ dynamics. Taken together, we envisage the chemical ecology of apple root-associated *S. ciscaucasicus* strain GS2 based on the obtained results.

## 2. Results

### 2.1. Phenotypic Characterization of S. ciscaucasicus GS2 under the Influence of Chemical OSMAC Approach by Alteration of Media Composition

**Figure 2:**
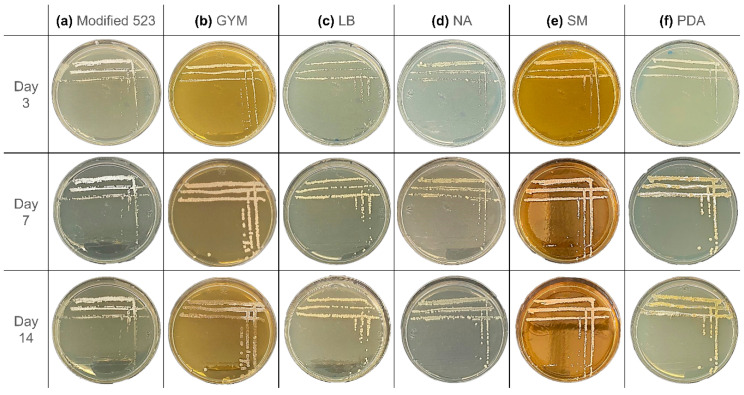
Chemical OSMAC approach-assisted phenotypic characteristics of *S. ciscaucasicus* GS2 grown on six different agar media for 14 days. (**a**) Modified 523 medium. (**b**) Glucose Yeast Malt medium (GYM). (**c**) Lysogeny medium (LB). (**d**) Nutrient agar (NA). (**e**) Streptomyces medium (SM). (**f**) Potato dextrose agar (PDA).

The first colonies of *S. ciscaucasicus* strain GS2 appeared on modified 523 agar after two days of cultivation, and by day three, all colonies were visible to the naked eye (Figure 2a). Between the third and seventh day of incubation, mild sporulation of the bacteria was observed. There was a decrease in sporulation from the seventh to fourteenth day, and the single colonies started to lose their color. The colonies presented a light beige color, which turned into white after sporulation. The colonies could be characterized as punctiform and have a smooth circular shape with a minor umbonate elevation. No alteration of the media color or odor was noted.

The morphology of *S. ciscaucasicus* on GYM agar (Figure 2b) was very different from that on modified 523 agar. Small colonies could already be observed by the third day of incubation, with a beige color and a smooth circular shape. By the seventh day of incubation, the colonies grew to 2–4 mm. The shape altered to an irregular form, and the surface became rather rugose. The colonies were characterized as umbonate. After 14 days of incubation, some parts of the bacterium lost their color and became transparent despite maintaining the same size, while some other parts started mild sporulation. The spores have a white color and are more intensive at the borders of the colonies. When touched with the loop, the colonies felt very brittle. Unlike that on modified 523 agar, the colonies grew only on the agar’s surface. No alteration of the media color or odor was noted.

The colonies on LB agar could be observed by day three (Figure 2c). After seven days of incubation, the colonies grew further; however, they remained punctiform. *S. ciscaucasicus* GS2 presented a light beige or yellow color on LB agar. There was a nominal growth of the colonies, but the diameter remained below 1 mm in size. No further changes were observed from day seven to the 14th day. The colonies had a circular shape with an umbonate elevation. The texture of the colonies was dry. The microorganism neither showed signs of sporulation, nor emitted odor, nor caused a change in the media color. In general, *S. ciscaucasicus* GS2 grew relatively slow on LB agar compared to the other tested media.

On NA, transparent colonies already grew by the third day of incubation (Figure 2d). Like modified 523 and LB agars, they have a light beige or yellow color and punctiform. After day 14 of incubation, the color became paler. Moreover, the texture became drier with time. The colonies had a circular shape with an umbonate elevation. No signs of sporulation, odors, and media color alterations were observed.

Tiny punctiform colonies of *S. ciscaucasicus* GS2 were visible on SM agar by day three, after which rapid growth of the colonies was noted (Figure 2e). The colonies oddly grew preferably in height than in diameter. By the seventh day, the colonies exhibited a round and highly pulvinated shape that had become flatter by day 14. By the third day, *S. ciscaucasicus* GS2 had a light beige or yellow color, which had slightly changed to beige or pinkish after seven days. The color did not change from the seventh to the fourteenth day. The colonies were dry and could be easily scraped from the agar plate with the loop. The bacteria did not show any sign of sporulation, did not emit odors, even though it caused darkening of the SM agar color.

The morphology of *S. ciscaucasicus* GS2 on PDA resembled its morphology on GYM agar (Figure 2f). Despite the tiny colonies, the culture could be observed by day three, showing a light beige or yellow color. After seven days of incubation, the color of the colonies turned yellow. There was also an increase in the size observed, with each colonial diameter at 2–3 mm. Apart from the size and color alterations, sporulation at specific parts of the culture was observed by the seventh day. By day 14, the sporulated colonies’ presented a darker yellow color. The rate of sporulation decreased by day 14 of incubation. The colonies of *S. ciscaucasicus* GS2 could be characterized as round or a bit irregular and with umbonate elevation. They were dry and could be easily scraped from the agar. Despite sporulation, no odor or change in the media color was observed.

### 2.2. Phenotypic Characterization of S. ciscaucasicus GS2 under the Influence of Biological OSMAC Approach by Dual-Culture Cultivation for Microorganismal Interaction

The biological OSMAC approach was employed by co-cultivating *S. ciscaucasicus* strain GS2 and endophytic *S. pulveraceus* strain ES16 on all six agar media used for the chemical OSMAC approach and monitored over 3, 7, and 14 days (Figure S1). The bacteria were co-cultivated by streaking each bacterium 1 cm apart, resulting in colony formation on modified 523 agar, LB agar, NA, and SM agar after three days. Noteworthy, no antagonistic interaction between the two bacteria was visible on these four media, given that they maintained their beige or white-colored irregular colonies throughout the incubation time, except for the slimy light browned *S. pulveraceus* ES16 colonies on modified 523 agar after 14 days. No odor or media pigmentation was observed, corroborating their respective phenotypes in axenic cultures. However, both species presented sporulation after seven days on PDA and GYM agar. The spore color of *S. pulveraceus* turned darker with time. This was also consistent with their phenotype in axenic cultures. After seven days of incubation, the bacterial colonies continued to grow over 14 days. Moreover, an alteration in the media color surrounding *S. pulveraceus* ES16 was observed, mainly on PDA, which was not the case for *S. ciscaucasicus* GS2.

*S. ciscaucasicus* GS2 was further co-cultivated with the pathogen *C. olidum* on PDA. Among the six tested media, *C. olidum* typically could grow only on PDA, which led us to use PDA for the dual-culture experiments with the pathogen. *S. ciscaucasicus* GS2 demonstrated sporulation on PDA during co-cultivation, which increased with time. After 14 days, *C. olidum* had grown freely and occupied the left side of each plate. However, its growth was limited on the plate’s right side due to colonization by *S. ciscaucasicus* GS2, even though an antagonistic interaction was not visible (Figure S2).

### 2.3. Production of Siderophores by S. ciscaucasicus GS2 in Axenic Culture and Structural Dereplication Using HPLC-HRMS^n^

**Figure 3:**
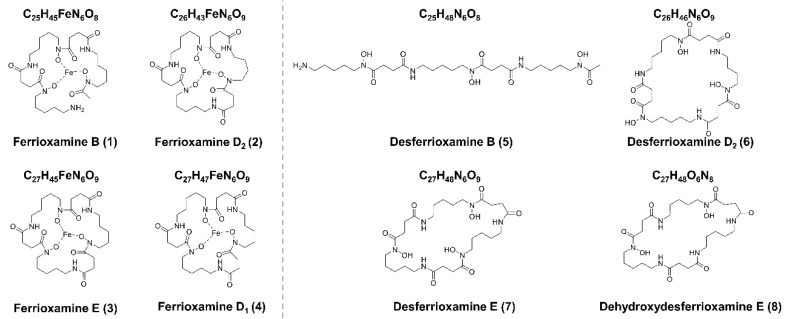
Chemical structures of the compounds reported in the present study.

Four different analogs of the siderophore family of ferrioxamines were produced by *S. ciscaucasicus* GS2 on PDA plates in axenic cultures over 14 days of incubation. These were ferrioxamine B (compound **1**), ferrioxamine D_2_ (compound **2**), ferrioxamine E (compound **3**), and ferrioxamine D_1_ (compound **4**) (Figure 3). Ferrioxamines B (**1**) and E (**3**) were also produced on GYM agar, but not compounds **2** and **4**. The production of compounds **2** and **4** on PDA started after three and seven incubation days, respectively. Generally, the intensities of the ferrioxamines increased with incubation time in the case of PDA. For example, after 14 days, the concentration of ferrioxamine E (**3**) was around 10-fold higher than ferrioxamine B (I ≈ 2.6E6 vs. I ≈ 6.7E5) (**1**), around 20-fold higher than ferrioxamine D_2_ (I ≈ 2.6E6 vs. I ≈ 7.7E4) (**2**), and almost the same as ferrioxamine D_1_ (**4**) (I ≈ 2.6E6 vs. I ≈ 2.2E6), even though the production of ferrioxamine D_1_ (**4**) started after seven days of incubation. Within one week, the concentration of ferrioxamine E (**3**) had a 20-fold increase (I ≈ 6.6E4 → I ≈ 2.6E6). The ferrioxamine concentrations on GYM agar mainly remained the same.

**Figure 4:**
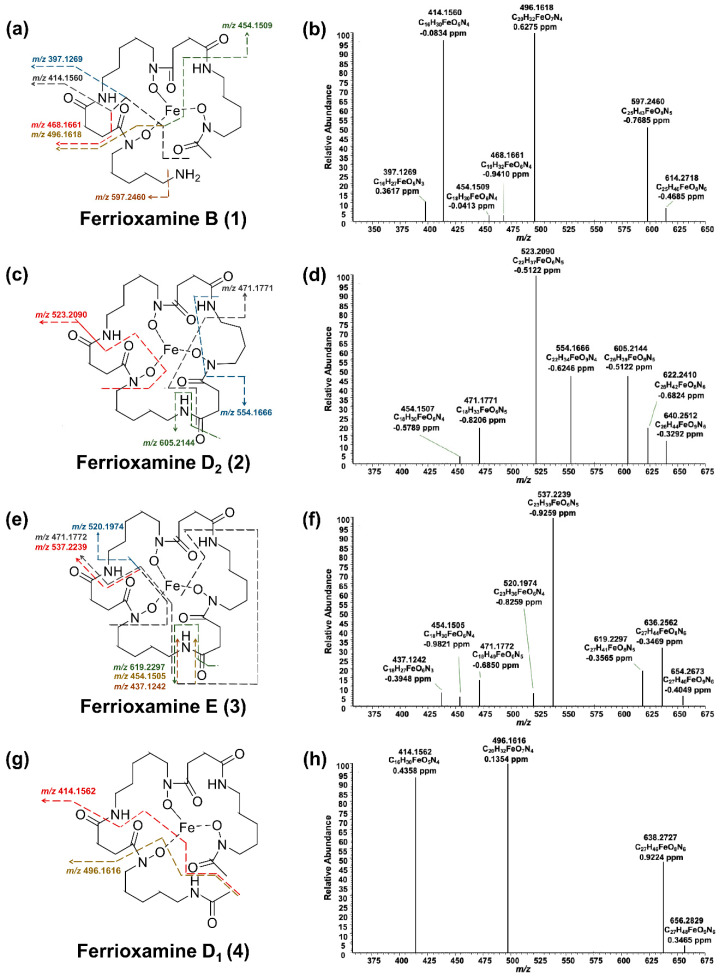
HPLC-HRMS^2^ analyses of ferrioxamines (**1**–**4**) produced by *S. ciscaucasicus* GS2. (**a**,**c**,**e**,**g**) The proposed mass spectral fragmentation pathway is annotated on each compound structure. (**b**,**d**,**f**,**h**) HRMS^2^ spectra of the ferrioxamines (**1**–**4**).

HRMS^2^ measurements were performed to identify the produced ferrioxamines (Figure 4). Due to the compounds’ robust and characteristic fragmentation by HRMS^2^ experiments, the compounds could be identified without performing further HRMS^3^ measurements. In the case of ferrioxamine B (**1**; Figure 4a,b), six main signals occurred during HRMS^2^, from which three had a higher relative abundance. The first signal, *m/z* 597.2460, was due to the cleavage of an amide group. The most abundant signal, *m/z* 496.1618, resulted from the loss of a water molecule along with a C_5_H_12_N_2_ chain. The second most abundant signal, *m/z* 414.1560, corresponded to the fragment C_16_H_30_FeO_5_N_4_. In all fragments, iron remained attached to the molecule. The HRMS^2^ analysis of ferrioxamine D_2_ (**2**; Figure 4c,d) resulted in five fragments. The fragment at *m/z* 605.2144 was due to water cleavage together with the cleavage of an amide group. The most abundant fragment *m/z* 523.2090 resulted from the cleavage of C_4_H_7_O_3_N. Another fragment yielding during HRMS^2^ was *m/z* 471.1771, corroborating C_18_H_33_FeO_6_N_5_. The difference between the structures of ferrioxamine E (**3**) and ferrioxamine D_2_ (**2**) is one methyl group (Figure 4c–f). Due to this reason, the HRMS^2^ fragmentation pattern followed by these compounds was similar. A cleavage of amide group and water moiety explained the fragment ion at *m/z* 619.2297. The peak with the highest intensity, *m/z* 537.2239, was also due to the loss of C_4_H_7_O_3_N, just as in the previous spectrum. The two spectra also shared the *m/z* 471.1772 fragment ion. Since ferrioxamine E (**3**) is a symmetrical molecule, the fragments could also be matched to a different part of the molecule than the one suggested above. Due to very low intensities of ferrioxamine D_1_ (**4**), HRMS^2^ measurement yielded only three fragments. It has an open cyclic structure, which resembles ferrioxamine B (**1**), thereby yielding the same fragmentation pattern (Figure 4g,h). The above fragmentation patterns were compared to the literature to confirm the structure of compounds **1**–**4** [33].

### 2.4. Production of Siderophores by S. ciscaucasicus GS2 during Dual-Culture Microorganismal Interaction and Structural Dereplication Using HPLC-HRMS^n^

**Figure 5:**
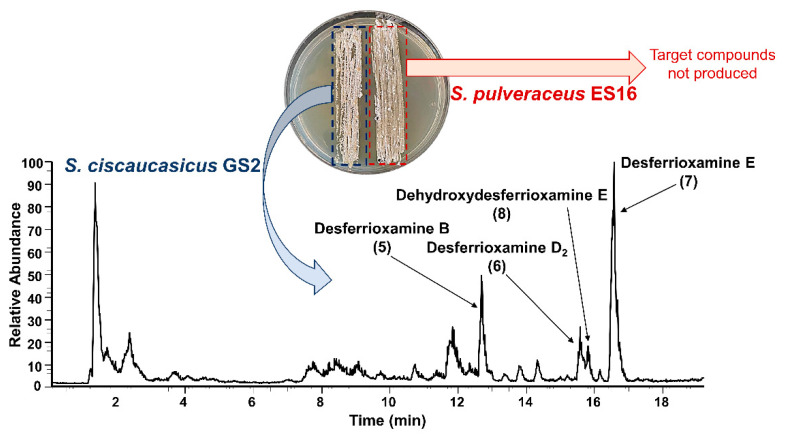
HPLC-HRMS analysis of the desferrioxamines (**5**–**8**) produced by *S. ciscaucasicus* GS2 during co-cultivation with *S. pulveraceus* ES16. The total ion current (TIC) showing compounds **5**–**8** produced by *S. ciscaucasicus* GS2 on PDA after three days of co-cultivation are presented. *S. pulveraceus* ES16 did not produce the target compounds both in axenic culture and during co-cultivation.

Although ferrioxamines (**1**–**4**) were only secreted on PDA by *S. ciscaucasicus* GS2 in axenic cultures, they were also produced by the organism on GYM agar during co-cultivation with endophytic *S. pulveraceus* ES16. Both on PDA and GYM agar, the production started after three days of co-cultivation. The intensities of ferrioxamines (**1**–**4**) were relatively higher during co-cultivation. Besides, on co-cultivated PDA plates, production of ferrioxamine D_1_ (**4**) started earlier (by day seven) compared to that of axenic cultures (after seven days). The concentration of ferrioxamines (**1**–**4**) on co-cultivated GYM agar plates was much higher than those in axenic cultures. Notably, the investigation of co-cultivated plates resulted in detecting several new compounds at very high abundances on PDA and GYM agar, which *S. ciscaucasicus* GS2 did not produce in axenic culture. A combination of selective microextraction of each organism from co-cultivated agar plates coupled to HRMS confirmed the production of these compounds by *S. ciscaucasicus* GS2 and not by *S. pulveraceus* ES16 (Figure 5). Using HRMS^2^ measurements, these compounds were classified as belonging to the siderophores group. Specifically, they were the unchelated ferrioxamines, known as De[s]ferrioxamines (compounds **5**–**8**, Figure 3). Desferrioxamines are small iron-chelating molecules which when bonded to a ferric ion, are referred to as ferrioxamines. The production of the desferrioxamines on the co-cultivated plates was detected after seven days of incubation on both PDA and GYM agar, and the compounds were produced more abundantly on PDA than on GYM agar.

**Figure 6:**
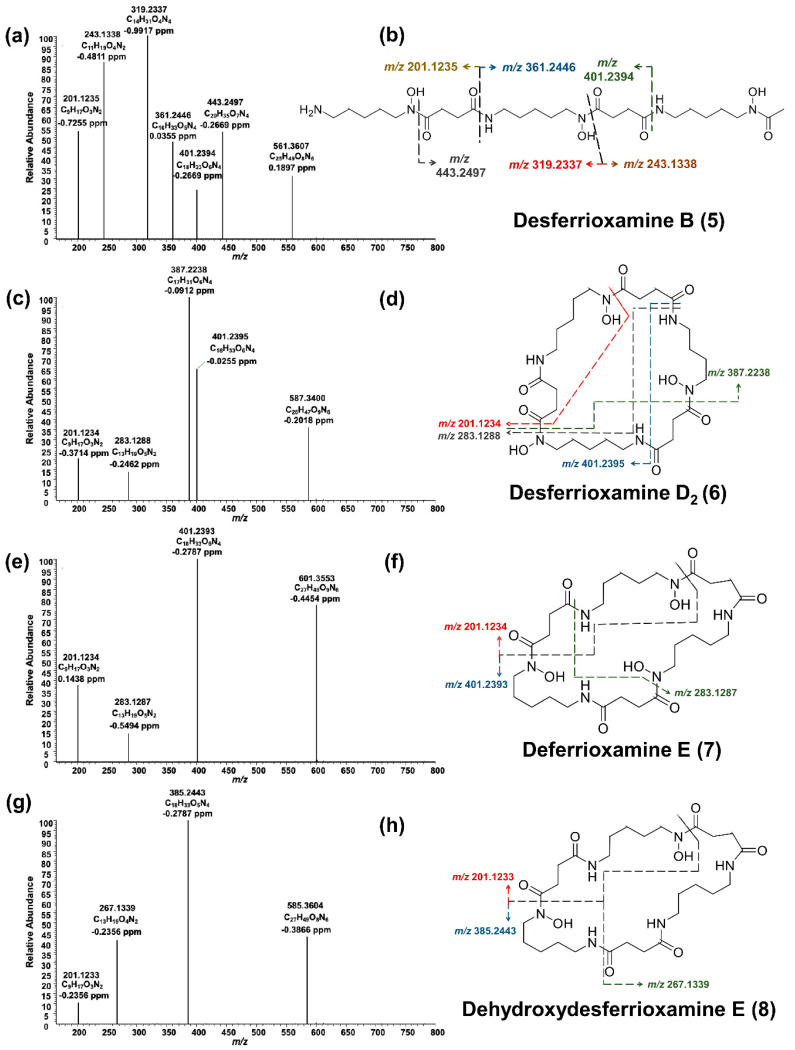
HPLC-HRMS^2^ analyses of desferrioxamines (**5**–**8**) produced by *S. ciscaucasicus* GS2. (**a**,**c**,**e**,**g**) HRMS^2^ spectra of the desferrioxamines (**5**–**8**). (**b**,**d**,**f**,**h**) The proposed mass spectral fragmentation pathway is annotated on each compound structure.

Desferrioxamine B (**5**) is primarily used as an iron-chelating agent in human medicine and is the only siderophore with a linear structure detected in the present work. HRMS^2^ measurement of this compound resulted in several specific fragments; the molecule fragmented between each weak nitrogen and carbon bond due to the oxygen double bond, which withdraws the electrons (Figure 6a,b). The most abundant signal was *m/z* 319.2337 from the cleavage of C_9_H_18_O_4_N_2_. The fragment ion *m/z* 201.1235 was common in all produced siderophores. Along with desferrioxamine B (**5**), the intensities of desferrioxamines D_2_ (**6**) and E (**7**) showed at least a 10-fold increase on PDA from the seventh to the fourteenth day (I ≈ 7.2E5 → I ≈ 3.4E6, I ≈ 3.8E5 → I ≈ 5.0E6, and I ≈ 1.6E5 → I ≈ 2.3E7). Since compounds **6**–**8** are cyclic, their fragmentation pattern was similar (Figure 6c–h). For example, resembling their ferrioxamine analogs, compounds **6** and **7** only have one methyl group difference. HRMS^2^ measurements of these metabolites resulted in three shared fragments, viz. *m/z* 401.2395, *m/z* 283.1288, and *m/z* 201.1234, as can be seen in their spectra. Apart from *m/z* 201.1233, the HRMS^2^ of **8** yielded two further fragments. The tandem HRMS spectra yielded for compounds **5**–**8** were verified by comparing the literature [34].

The co-cultivation of *S. ciscaucasicus* GS2 and pathogenic *C. olidum* was only carried out on PDA (Figure S2), during which *S. ciscaucasicus* GS2 produced all four ferrioxamines (**1**–**4**). Interestingly, their production started already by the third day of co-cultivation and could be detected at very high intensities (I ≈ E5–E6 by the third day). Apart from dihydroxy desferrioxamine E (**8**), all other desferrioxamines (**5**–**7**) were not produced (<LOD) during co-cultivation with *C. olidum*. Dehydroxydesferrioxamine E (**8**) production—the only desferrioxamine produced in minute amounts in axenic culture—was also observed by the third day during co-cultivation, pending a decrease in its concentration over 14 days.

### 2.5. Trend and Dynamics of Siderophore Production by S. ciscaucasicus GS2 under the Influence of Chemical and Biological OSMAC Triggers

*S. ciscaucasicus* GS2 started producing ferrioxamine B (**1**) already by the third day in axenic culture and all co-cultivations on GYM agar and PDA. On the third day, the amount of ferrioxamine B (**1**) produced by *S. ciscaucasicus* GS2 during co-cultivation with *C. olidum* was around 30-fold the amount produced during co-cultivation with *S. pulveraceus* ES16 (I ≈ 2.0E6 vs. I ≈ 2.7E3), and around 20-fold the amount in axenic culture (I ≈ 2.0E6 vs. I ≈ 1.9E4). After 14 days, the intensity of compound **1** was around 10-fold higher on PDA when co-cultivated with *S. pulveraceus* ES16 compared to that in axenic culture and co-cultivation with *C. olidum* (I ≈ 1.4E6 vs. I ≈ 7.0E5 and vs. I ≈ 2.8E5). From the seventh day through to the fourteenth day, there was a slight decrease of ferrioxamine B (**1**) produced on PDA (I ≈ 2.8E6 → I ≈ 1.4E6). This pattern was not observed on GYM agar.

Ferrioxamine D_2_ (**2**) was not produced by *S. ciscaucasicus* GS2 in axenic culture on GYM agar but was detected during co-cultivation with *S. pulveraceus* ES16. While on PDA, it was produced in both axenic and co-cultivations with both the endophyte and the pathogen. By day three of incubation, ferrioxamine D_2_ (**2**) was still not detected in axenic culture and during co-cultivation with *S. pulveraceus* ES16, but was detected with relatively high intensity (I ≈ 2.9E5) during co-cultivation with the pathogen *C. olidum*. By the 14th day, the amount of compound **2** produced on PDA was almost 10-fold compared with co-cultivation with *S. pulveraceus* ES16 and *C. olidum* and axenic cultures. Moreover, an around 10-fold decrease in the intensity of compound **2** was observed from the third to the seventh day in *C. olidum* co-cultivation studies that stabilized later on (I ≈ 2.9E5 → I ≈ 2.8E4 → I ≈ 7.8E4).

Ferrioxamine E (**3**) was the most abundant siderophore produced by *S. ciscaucasicus* GS2, which was detected by the third day of incubation under all tested conditions. During co-cultivation with *C. olidum*, the bacterium started producing compound **3** at high amounts (I ≈ E6), which remained the same throughout the 14 days of observation, while in axenic cultures and during co-cultivation with *S. pulveraceus* ES16, the intensities were 20-fold less (I ≈ 4.47E6 vs. I ≈ 5.3E4 and vs. I ≈ 2.1E4) on the third day and increased with time (to I ≈ E6). The production of compound **3** after 14 days during co-cultivation with *S. pulveraceus* ES16 on GYM agar was higher than on PDA (I ≈ 1.0E7 vs. I ≈ 7.0E6). In axenic culture on GYM agar, the intensity of compound **3** remained in the range of E4 through the entire incubation time, but an increase was observed in axenic culture on PDA (I ≈ 5.3E4 → I ≈ 6.6E4 → I ≈ 2.6E6).

Ferrioxamine D_1_ (**4**) was not produced by *S. ciscaucasicus* GS2 in axenic culture on GYM agar but could be detected after 14 days on PDA with an intensity of (I ≈ 2.2E6). Low quantities of compound **4** were observed on the third day during co-cultivation with *S. pulveraceus* ES16 on GYM agar (I ≈ 8.2E3), while it could not be detected (<LOD) on PDA after three days of co-cultivation. Compound **4** was first observed by the seventh day of incubation that persisted over 14 days on the seventh and fourteenth day on PDA (I ≈ 4.4E5 and I ≈ 4.8E5). However, co-cultivation studies on GYM agar showed higher amounts of compound **4** over the same period (I ≈ 1.0E6 → I ≈ 1.9E6). Besides, ferrioxamine D_1_ (**4**) was observed already after the third day during co-cultivation with *C. olidum*, and the compound intensity slightly increased over 14 days (I ≈ 9.9E4 → I ≈ 4.2E5 → I ≈ 4.8E5).

Desferrioxamine B (**5**) was not produced by *S. ciscaucasicus* GS2 in axenic cultures on any tested media and during co-cultivation with *C. olidum*. It was only produced during co-cultivation of the bacterium with *S. pulveraceus* ES16. The production was first observed by the seventh day on both PDA and GYM agar. From the seventh to the fourteenth day, there was a slight decrease in the compound intensity on GYM agar (I ≈ 1.6E6 → I ≈ 7.4E5), whereas on PDA, an increase was observed (I ≈ 7.2E5 → I ≈ 3.4E6). Desferrioxamine D_2_ (**6**) was also not produced by *S. ciscaucasicus* GS2 in axenic cultures on any tested media and during co-cultivation with *C. olidum*, similar to compound **5**. It was only produced during co-cultivation with *S. pulveraceus* ES16 on PDA and GYM agar. The production pattern of compound **6** was similar to compound **5** in that it was first detected by the seventh day on both media. From the seventh to the fourteenth day, there was a slight decrease in the compound intensity on GYM agar (I ≈ 1.4E5 → I ≈ 8.3E4), whereas on PDA, an increase was observed (I ≈ 3.8E5 → I ≈ 5E6).

Desferrioxamine E (**7**) was not produced by *S. ciscaucasicus* GS2 in axenic cultures on GYM agar and during co-cultivation with *C. olidum*, but was detected on the 14th day in axenic culture on PDA at very low intensities (I ≈ 9.5E3). However, the production of compound **7** started during co-cultivation with *S. pulveraceus* ES16 by the seventh day on both PDA and GYM agar. While the compound intensity remained around E6 on GYM agar from the seventh through to the 14th day (I ≈ 3.0E6 and I ≈ 1.2E6), there was around a 10-fold increase in its intensity during co-cultivation with *S. pulveraceus* ES16 on PDA (I ≈ 1.6E6 → I ≈ 2.3E7). Noteworthy, desferrioxamine E (**7**) and ferrioxamine E (**3**) were the two main siderophores produced by *S. ciscaucasicus* GS2.

Dehydroxydesferrioxamine E (**8**) was not produced by *S. ciscaucasicus* GS2 in axenic culture on GYM agar but was detected in axenic culture after 14 days on PDA (I ≈ 7.4E4). Strikingly, compound **8** is the only desferrioxamine produced by the bacterium during co-cultivation with *C. olidum*. Its production already started by the third day, which decreased with time and stabilized over 14 days (I ≈ 2.6E5 → I ≈ 4.6E3 → I ≈ 3.0E3). The intensity of compound **8** during co-cultivation with *S. pulveraceus* ES16 on GYM agar was noted around E5, whereas it was slightly higher on PDA (I ≈ 6.3E5 vs. I ≈ 4.7E6). From the seventh to the 14th day, there was a very slight increase observed in both cases.

## 3. Discussion

### 3.1. Siderophore-Mediated Communication between Different Microbial Species Belonging to the Same Genus: Mutualistic Cooperation or Piracy?

Iron is a vital element for all organismal growth since it is essential for various processes such as catalytic activities, DNA and RNA synthesis, and photosynthesis [35,36]. Their uptake is enabled via a group of chemically diverse small organic molecules known as siderophores, with receptors that have a high affinity for the valuable ferric ion. Numerous organisms are capable of secreting specific siderophores in order to scavenge iron from their environment. Thus far, Kramer et al. (2020) has described siderophores as “public goods.” The producing organisms can either reabsorb the siderophores or diffuse away and be unreachable to their original producers, thereby benefiting neighboring organisms of the same or similar genera possessing the same recognition receptor(s) [37,38]. In particular, the uptake of siderophores in a given niche depends on whether the producing organism and the neighboring organisms share mutual recognition receptor(s) for the siderophore [37,39]. For instance, a Gram-negative bacteria’s outer cell membrane is equipped with a β-barrel receptor for specific recognition, which undergoes a conformational change once bound to a specific siderophore, thus transferring the complex to the periplasm [40,41]. On the other hand, Gram-positive bacteria do not have such a membrane, and specific ATP-binding cassette transporters import the siderophores across the cell [39,40,41]. Therefore, selected siderophores produced by one organism are shared with other neighbor microorganisms belonging to the same genera. For instance, *S. lividans* and *S. viridosporus* can uptake ferrioxamine B and ferrioxamine G, respectively, despite their inability to produce these siderophores [42]. Another study demonstrated how the production of desferrioxamine E by *S. griseus* played a role in stimulating the morphology, growth, development, and secondary metabolite formation of associated *S. tanashiensis* [43]. It is now evident that siderophores play a role in mediating interactions in the rhizobium, and their secretion can be characterized as mutualistic cooperation or antagonism depending on their reabsorption and biological effects [39].

The enhanced and triggered production of siderophores (**1**–**8**) by *S. ciscaucasicus* GS2 during interaction with co-cultivated *S. pulveraceus* ES16 points to a siderophore-mediated communication between them. Notably, *S. ciscaucasicus* GS2 did not antagonize *S. pulveraceus* ES16 on any tested media (Figure S1). Moreover, disc diffusion assays revealed that extracts of *S. ciscaucasicus* GS2 containing produced siderophores did not demonstrate antimicrobial or antagonistic activity against *S. pulveraceus* ES16 (Figure S3). Taking cues from literature in combination with our present results, this suggests that other coexisting *Streptomyces* species in roots or soil could also consume the siderophores produced by *S. ciscaucasicus* GS2. Concomitantly, it can be suggested that these siderophores might influence the enrichment of *Streptomyces* species observed in recent studies [9,26,27,28] during dysbiosis of the root-associated and soil microbiome in the case of ARD.

However, microbial piracy during the complex cascade of interactions in the environment cannot be ruled out. As demonstrated by Galet et al. (2015), for example, *Pseudomonas fluorescence* pirates ferrioxamines produced by *Streptomyces* species since it possesses the *FoxA* membrane receptor that enables selective uptake of ferrioxamines and the hydroxamate siderophore, ferricoelichelin [44]. A plethora of microorganisms encompassing various genera and species interact and communicate in both healthy and ARD soils [5,7,45]. Therefore, it is also feasible that apart from siderophore-mediated mutualism between *S. ciscaucasicus* GS2 and associated *Streptomyces* species, these molecules might also influence the growth, virulence, or selection of other pathogenic microorganisms equipped with a fitting membrane receptor(s) for the produced siderophores.

### 3.2. Siderophore-Mediated Microbial Communication at the Root-Soil Interface

Apart from solubilizing Fe^3+^ ions, in conditions with low iron bioavailability, iron-chelated siderophores such as ferrioxamines may assist the uptake of iron by the apple tree [35,46]. The uptake of hydroxamate and catecholate siderophores produced in the rhizobium by plants has been previously reported [47,48]. While the exact mechanism remains unknown, two plausible mechanisms were proposed. One study hypothesizes that the iron-chelated siderophores are transferred to the plant root apoplast, followed by a reduction of the siderophores, thereby leading to the donation of Fe^2+^ ions to the plant’s transport system [35,49]. However, Masalha et al. (2000) suggested that microbial siderophores can undergo ligand exchange with phytosiderophores, a mechanism that relies on various parameters [50]. Almost four decades ago, Kloepper et al. (1980) introduced the biocontrol potential of *Pseudomonas* species by producing a class of siderophores known as pyoverdines [51]. Since then, several systematic studies investigating the role of microbial siderophores against plant diseases have been carried out. For instance, Ghazy and El-Narawy (2020) recently associated the biocontrol potential of *Bacillus subtilis* and *Pseudomonas koreensis* against *C. maydis* induced in late wilt disease through their siderophore production [52]. Therefore, siderophores can be considered competitive agents among the microbiota, implying that microorganisms not producing them cannot participate in the competition and, therefore, undergo iron starvation [53]. As demonstrated herein by co-cultivation with *C. olidum*, by producing siderophores such as desferrioxamines, microorganisms such as *S. ciscaucasicus* GS2 can decrease the bioavailability of Fe^3+^ ions in soil by suppressing and depriving other pathogens with diverse recognition receptors for this essential ion. On the one hand, our results suggest that on sensing the presence of *C. olidum* in its vicinity, *S. ciscaucasicus* GS2 aggressively starts producing ferrioxamines as a defense mechanism. On the other hand, it might be that a decrease of Fe^3+^ ion concentration accompanies *C. olidum* in the same media (nutrient pool) since it also uses them up for its growth and sustenance, leading to the early production of ferrioxamines by *S. ciscaucasicus* GS2. A decrease in the bioavailability of Fe^3+^ ions is linked to the virulence suppression of phytopathogens [53,54,55]. The role of siderophores produced by *S. ciscaucasicus* GS2 on the virulence exhibited by *Cylindrocarpon*-like fungi, other ARD-related phytopathogens, or the effect on host apple plants should be investigated further.

### 3.3. Siderophore-Mediated Microbial Contribution to Plant Immunity via Induced Systemic Resistance

Besides iron-chelating properties, siderophores have been reported as elicitors of induced systemic resistance (ISR), a typical plant defense mechanism activated by infection. Other elicitors of this mechanism include volatile organic compounds (VOCs) and microbe-associated molecular patterns (MAMPs) [53]. ISR was discovered when researchers observed plant health promotion with beneficial bacteria colonizing its roots [53,56]. Previous studies have suggested that these beneficial rhizobacteria manipulate the plant hormone signaling pathways through selected elicitors’ production [57]. However, to elicit ISR, a minimal bacterial concentration of about 10^5^ to 10^7^ cells per gram of root is necessary [53,58]. This population of rhizobacteria and siderophore secretion can cause Fe^3+^ ion deficiency in the soil, depriving the plant of their uptake and inducing plant root responses, for example, acidification of the rhizosphere, development of root hairs, and increased ferric reductase activity [53]. Besides, Aznar et al. (2014) reported activation of an immune response by iron scavenging and the vital role of the heavy metal homeostasis in plants [36]. They specifically proved that desferrioxamines activated immunity markers of *Arabidopsis* and protected it against the pathogenic *P. syringae*. The iron-containing siderophores, however, did not show a positive effect on the plant. Numerous other examples of siderophore-mediated ISR activation have been reported. For example, the presence of the siderophore, pyochelin, produced by *P. aeruginosa* strain 7NSK2 reduces the damping-off caused by the pathogen *Pythium splendens* [59]. *P. fluorescens* strain WCS374r produced the siderophore pseudobactin, thereby triggering ISR in rice infected with *Magnaporthe oryzae* [60,61]. Against this background, it is interesting to note the production and modulation of the iron-chelated siderophores ferrioxamines (**1**–**4**) as well as the unchelated desferrioxamines (**5**–**8**) by *S. ciscaucasicus* GS2 under different chemical and biological OSMAC conditions. It is plausible that they can cause a deficiency of bioavailable iron in the apple plant rhizosphere in the field, triggering ISR in the plants. This might either aid or negatively influence the plants for coping with different biotic stresses presented during the incidence of ARD, which should be studied further.

Our results provide a scientific handle to study further the chemical basis of *S. ciscaucasicus* strain GS2 (and other *Streptomyces* species) in the greenhouse and field setups to understand the organism’s biochemical role. Further studies, for example, by employing labeled siderophores or their precursors coupled to the HRMS^n^ assisted mass-balance approach and mass spectrometry imaging experiments in the greenhouse and field setups, can trace the flow of metabolites across the root-microbe-soil continuum, leading to a definitive understanding of the in situ chemical ecology of the produced siderophores, as well as other *Streptomyces* metabolites concerning ARD.

## 4. Materials and Methods

### 4.1. Microorganisms Used in the Present Study

Endophytic *Streptomyces ciscaucasicus* strain GS2 was isolated from unsterilized apple plant roots (*Malus domestica*, rootstock M26; grown in soil from reference site Ruthe, plot 4.7 with grass cover, 2018) within the ORDIAmur Biotest Central Experiment setup 2 (CE2) of the BMBF BonaRes program. For isolation, root cortex cells containing actinobacteria structures were microscopically identified [9] (FUN staining, fluorescence microscopy, FUN 1® Cell stain, F-7030, Molecular Probes^TM^, Thermo Fisher Scientific, Life Technologies, USA), tissue cubes of approximately 2 mm^3^ volume were cut out, immediately transferred to sterile water, carefully agitated, and aliquots of the liquid plated in dilution series on R2A medium. Microscopic screening of single colonies and selection of actinobacteria isolates proceeded to subculture on R2A or later alternatively on malt extract agar (MEA, 2%). Endophytic *Streptomyces pulveraceus* strain ES16 was isolated from surface-disinfected apple roots (*Malus domestica*) grown in grass control (ARD-unaffected) soil within the Central Experiment setup 1 (CE1; Ellerhoop, spring 2018) of the BMBF BonaRes ORDIAmur program [12]. For isolation, 1 cm pieces of surface-disinfected fine roots (Ø < 2 mm) were placed in Petri dishes containing 523 medium [62] and stored for approximately seven days at room temperature. For both isolates, emerging colonies were picked and incubated for one to seven days in liquid 523 medium until growth was visible. 1 mL of suspension was used for DNA extraction based on Quambusch et al. (2014) [63]. The 16S rRNA gene was amplified using the primers 27f (AGAGTTTGATCCTGGCTCAG) and 1492r (GGYTACCTTGTTACGACTT) [64]. Moreover, 16S rDNA fragments were sequenced with the Sanger method (Sanger et al. 1977) [65] by Microsynth Seqlab (Göttingen, Germany), and the obtained sequence was blasted (Blastn) [66] against the NCBI database (https://www.ncbi.nlm.nih.gov/, accessed on 15.08.2018), and submitted (accession number MW580673.1). The phytopathogen *Cylindrocarpon olidum* (Wollenw.) Wollenweberwas (DSM No. 62520; neotype strain) was obtained from Leibniz Institute DSMZ, German Collection of Microorganisms and Cell Cultures GmbH, Braunschweig, Germany. The cultivation, long-term storage, and working culture maintenance were performed according to DSMZ guidelines.

### 4.2. OSMAC-Assisted Cultivation and Fermentation

We optimized our chemical OSMAC approach established for endophytic bacteria [67] to cultivate *S. ciscaucasicus* GS2. Thus, the endophyte was cultivated in six different attuned media conditions, ranging from nutrient-rich to minimal conditions, for selecting different phenotypes (e.g., sporulation under stress) and metabolic expression patterns, both under submerged conditions (broth) as well as on agar. These included modified 523 medium, Glucose Yeast Malt medium (GYM), Lysogeny medium (LB), nutrient medium (agar, NA; broth, NB), potato dextrose medium (agar, PDA; broth, PDB), and streptomyces medium (SM). The OSMAC conditions, physio-chemical triggers, and selection pressures presented by each of these six media were recently published [68]. The components of the media are detailed in Table S1. The cultures were incubated at 28 °C (Memmert Incubator, Schwabach, Germany), and the bacterial growth, morphology, and OSMAC-relevant phenotypic characteristics were monitored and documented regularly over 14 days.

The biological OSMAC approach was employed by dual culture co-cultivation [32] with *S. pulveraceus* strain ES16 and the phytopathogen *C. olidum* strain DSM 62520. Half-loopful *S. ciscaucasicus* GS2 and half-loopful *S. pulveraceus* ES16 were streaked on agar plates with a distance of 1 cm apart from each other for all six media in triplicates. The plates were incubated at 28 °C and observed for 3, 7, and 14 days, respectively. While after three days, the organisms had shown colony formation and steady growth on modified 523, NA, LB, and SM, only minimal or no growth was observed on PDA and GYM agar during co-cultivation with this inoculation approach. Given that both bacteria form colonies in their axenic cultivations on both PDA and GYM agar, to overcome the above problem, we decided to increase the inoculum load (i.e., a higher amount of each bacterium) for co-cultivation on these two media. While maintaining the 1 cm distance, one loopful of each bacterium was streaked back and forth to form a 2 cm thick seeding on each agar plate, which led to the growth of the organisms on PDA and GYM agar during co-cultivation. For the axenic culture of *C. olidum*, consistent growth was only observed on PDA among the tested media. Therefore, we performed the co-cultivation of *S. ciscaucasicus* GS2 and *C. olidum* only on PDA. For this, small agar plugs of *C. olidum* were suspended in 1 mL of sterile, double-distilled water. A total of 100 μL of the emulsion was then added to the PDA plate, which was spread with a sterile swab to form a 2 cm thick culture seed. The inoculation of *S. ciscaucasicus* GS2 was carried out with the same procedure as above. The triplicates were observed for 3, 7, and 14 days, respectively.

For submerged cultivation and fermentation, 500 mL broth of each of the six media was prepared in 1000 mL Erlenmeyer flasks and autoclaved at 121 °C for 15 min (Autoclave VB-55, Systec, Wettenberg, Germany). Bacteria cultivated on agar media was added to the broth under sterile conditions, and the flask was sealed tightly. The inoculated broths were placed in a shaker incubator (Lab-Rotation Incubator Multitron 2, INFORS HT, Einsbach, Germany) and fermented at 125 rpm at 28 °C. As negative controls, 100 mL of all six sterile, uninoculated media broths were incubated simultaneously.

### 4.3. Extraction of Agar Plates

The 3-, 7- and 14-day old bacterial agar plates and negative control plates were crushed to small pieces using a spatula. Each plate was covered with 20 mL HPLC-grade MeOH (J. T. Baker, Deventer, The Netherlands) and was mixed well. The mixture was transferred to a beaker and was extracted in an ultrasonic bath (Sonorex Longlife, Bandelin, Berlin, Germany). After 15 min, the mixture was filtered. This step was repeated thrice (i.e., extraction with 3 × 20 mL fresh MeOH). The filtrate was concentrated to dryness under reduced pressure in a rotary evaporator (Laborota 4001 Efficient, Heidolph, Schwabach, Germany) and resuspended in 4 mL HPLC-grade MeOH (J. T. Baker, Deventer, The Netherlands) for further analyses.

### 4.4. Extraction of Fermented Cultures (Broths)

On the third, seventh, and fourteenth day of cultivation and under sterile conditions, 100 mL of each inoculated broth was transferred to a 250 mL Erlenmeyer flask. Each flask was placed in an ice bath and ultrasonicated thrice for 15 min in the Sonoplus ultrasonic device (Bandelin, Berlin, Germany) equipped with ultrasonic lance UW 3200 (Bandelin, Berlin, Germany). The flasks’ content was transferred to Eppendorf centrifuge tubes and was centrifuged at 10,000 rpm for 10 min to separate the biomass (Centrifuge Allegra™ IR, Beckman Coulter GmbH, Krefeld, Germany). The supernatant was decantated, frozen, and was freeze-dried overnight in a Vaco 5 freeze dryer (ZIRBUS technology GmbH, Bad Grund, Germany). The residue was extracted thrice with 20 mL HPLC-grade MeOH (J. T. Baker, Deventer, The Netherlands) in an ultrasonic bath (Sonorex Longlife, Bandelin, Berlin, Germany) for 15 min, and filtered. The filtrate was concentrated to dryness under reduced pressure in a rotary evaporator (Laborota 4001 Efficient, Heidolph, Schwabach, Germany), and was resuspended in 4 mL HPLC-grade MeOH (J. T. Baker, Deventer, The Netherlands). The uninoculated negative control blanks were extracted following the same procedure.

### 4.5. High-Performance Liquid Chromatography-High Resolution Tandem Mass Spectrometry (HPLC-HRMS^n^)

A total of 100 μL of each extract was transferred to an HPLC vial and was concentrated to dryness in a concentrator (Savant SDP1010 SeedVac, Thermo Fisher Scientific, Waltham, MA, USA). A total of 100 μL of a 2:1 H_2_O and MeOH mixture was added to the vial, and the residue was reconstituted by vortexing (Vortex mixer VF2, IKA-Werke GmbH, Staufen, Germany). The sample was centrifuged at 6600 rpm for 5 min (Mini Centrifuge, MCF-2360, LMS CO., LTD., Tokyo, Japan), and the supernatant was transferred to an inlet and measured. The measurements were either carried out with an Agilent 1200 system HPLC (Waldbronn, Germany) coupled to an LTQ-Orbitrap XL mass spectrometer (Thermo Fisher Scientific, Waltham, USA) or a Nexera X2 HPLC (Shimadzu Scientific Instruments, Columbia, MD, USA) coupled with an LTQ-Orbitrap mass spectrometer (Thermo Fisher Scientific, Waltham, USA). A Nucleoshell C18 reverse-phase column (2.7 μm, 150 × 4.6 mm, Macherey-Nagel, Düren, Germany) was used for chromatographic separation at 30 degrees with H_2_O (+ 0.1% HCOOH) (A) and MeOH (+ 0.1% HCOOH) (B) gradient (flow rate 300 μL min^−1^). The gradient program was as follows: 95% A isocratic for 2 min, linear gradient to 100% B over 26 min, 100% B isocratic for 6 min, the system returned within 0.5 min to initial conditions of 95% A and was equilibrated for 2.5 min. The LTQ-Orbitrap XL and the LTQ-Orbitrap were equipped with a HESI ion source with 5 kV voltage at 350 °C. The ion source was operated with He as collision gas, and N_2_ as sheath- (40 arbitrary units), and auxiliary gas (8 arbitrary units). The spectrometers were operated in positive modes with a mass range of *m/z* 100–1600 with a nominal mass resolving power of 60,000 at *m/z* 400 with a scan rate of 1 Hz, with the internal lock masses N-butylbenzenesulfonamide ([M+H]^+^ *m/z* 214.0896) and dibutyl phthalate ([M+H]^+^ *m/z* 279.1591). The analyses were performed using Xcalibur software v. 2.2 SP1.48. (Thermo Scientific, Bremen, Germany). The acquired masses were sorted by intensity (I > 1.00E3). A maximum mass tolerance of 2 ppm was accepted. For structure elucidation, tandem HRMS experiments were performed with the LTQ-Orbitrap mass spectrometer with collision-induced dissociation (CID) energies of 15, 25, and 35 eV. SciFinder, Knapsack, and PubChem were used as reference databases. After structures of the target compounds were elucidated, we evaluated the trends (dynamics) of production of these compounds under the influence of the tested OSMAC conditions. For this, the compound masses were first sorted by peak intensity (I > 1.00E3), keeping within the maximum mass tolerance of 2 ppm using Xcalibur software v. 2.2 SP1.48. (Thermo Scientific, Bremen, Germany), and further exported to an Excel datasheet (Version 16.45, Microsoft Office 365) for comparison and graphical representation. For better visualization of the data, the obtained compound peak intensities were calculated and represented on a logarithmic scale (log_10_ base) to compare very high and low intensities of selected compounds produced under different conditions, using Microsoft Excel (Version 16.45, Microsoft Office 365).

### 4.6. Disk Diffusion Assays to Test Siderophore-Mediated Antagonism of S. pulveraceus Strain ES16

*S. pulveraceus* ES16 cultures were prepared by adding 100 μL of the bacterial dispersion to agar plates. Using a sterile swab, the cultures were spread across the plate (spread plate technique). The plates were left in the fridge for 3–4 h. In the meantime, the pooled extract of *S. ciscaucasicus* strain GS2 was prepared by mixing 100 μL of each 14-day bacterial extract, which was then used to prepare a dilution series of 0%, 20%, 40%, 60%, 80%, and 100% of the extract using MeOH. After that, 20 μL of each solution was loaded onto sterile filter paper disks (MN 827 ATD filter paper circles, 6 mm diameter, Macherey-Nagel GmbH & Co. KG, Düren, Germany). The disks were placed carefully on the seeded plates, incubated at 28 °C, and observed over 48 h for the zone of inhibitions (ZOIs) around the discs.

## Data Availability

The data presented in the study are included in the SupplementaryMaterial, further inquiries can be directed to the corresponding author.

## Supplementary Materials

The following are available online. Table S1. The composition of the six media prepared for this study using the OSMAC approach. Figure S1. Chemical OSMAC approach-assisted phenotypic characteristics of *S. ciscaucasicus* GS2 co-cultivated with *S. pulveraceus* ES16 on six different agar media for 14 days. Figure S2. Chemical OSMAC approach-assisted phenotypic characteristics of *S. ciscaucasicus* GS2 co-cultivated with *C. olidum* on potato dextrose agar for 14 days. Figure S3. Disc diffusion assay to enumerate the antimicrobial or antagonistic efficacies of extracts of *S. ciscaucasicus* GS2 containing the produced siderophores against *S. pulveraceus* ES16.

## Sample Availability

*S. ciscaucasicus* strain GS2 and *S. pulveraceus* strain ES16 are available from G.G.-S. and F.M.-D., respectively, upon request.
