# Supplementary material for: Production of Siderophores by an Apple Root-Associated Streptomyces ciscaucasicus Strain GS2 Using Chemical and Biological OSMAC Approaches
Source: Molecules. 2021 Jun 9;26(12):3517. doi: 10.3390/molecules26123517 (PMC8228313; doi:10.3390/molecules26123517)
Supplement: Supplementary file 1 [file molecules-26-03517-s001.zip › molecules-1234109-supplementary.pdf]

SUPPLEMENTARY MATERIAL

Production of siderophores by an apple root-associated *Streptomyces ciscaucasicus* strain GS2 using chemical and biological OSMAC approaches

Reyhaneh Armin[1,†], Sebastian Zühlke[1], Gisela Grunewaldt-Stöcker[2], Felix Mahnkopp-Dirks[3], Souvik Kusari[1]*

[1] Center for Mass Spectrometry (CMS), Faculty of Chemistry and Chemical Biology, Technische Universität Dortmund, Otto-Hahn-Str. 6, 44227 Dortmund, Germany

[2] Institute of Horticultural Production Systems, Section Phytomedicine, Leibniz Universität Hannover, Herrenhäuser Str. 2, 30419 Hannover, Germany

[3] Institute of Horticultural Production Systems, Section Woody Plant and Propagation Physiology, Leibniz Universität Hannover, Herrenhäuser Str. 2, 30419 Hannover, Germany

[†] Present address: Environmental Analysis, Currenta GmbH & Co. OHG, Chempark Blg Q18, 51368 Leverkusen, Germany; and Instrumental Analytical Chemistry (IAC) and Centre for Water and Environmental Research (ZWU), University of Duisburg-Essen, Universitätsstrasse 5, 45141 Essen, Germany

***** Correspondence: souvik.kusari@tu-dortmund.de; Tel.: +49-2317554086 (S.K.)

Table S1. The composition of the six media prepared for this study using the OSMAC approach, viz. modified 523 medium, glucose yeast malt medium (GYM), lysogeny medium (LB), nutrient medium (agar, NA; broth, NB; HiMedia, Mumbai, India), potato dextrose medium (agar, PDA; broth, PDB; HiMedia, Mumbai, India), and streptomyces medium (SM). SM was procured as an optimized media for cultivating *Streptomyces* species from Sigma Aldrich (Id. 85883, Lot BCBZ5408, St. Louis, USA; exact media composition not disclosed by the company). For solid media, 10 g/L of granulated agar (Difco^{TM}, Becton, Dickinson and Company, New Jersey, USA) was added prior to autoclaving.

**Components** (source)	**Amount (g/L)**
**Modified 523 medium** (prepared in the lab; composition optimized from Viss, P., Brooks, E. and Driver, J. (1991). A simplified method for the control of bacterial contamination in woody plant tissue culture. *In Vitro Cell Dev. Biol.* 27, 42. doi: 10.4103/0973-1296.96547)	
Saccharose (Sigma-Aldrich, St. Louis, USA, CAS: 57-50-1)	10
Peptone from Meat (Sigma-Aldrich, St. Louis, USA, CAS: 91079-38-8)	8
Yeast Extract (Sigma-Aldrich, St. Louis, USA, CAS: 8013-01-2)	4
KH_2PO_4 (Sigma-Aldrich, St. Louis, USA, CAS: 7778-77-0)	2
$MgSO_4 x7H_2O$ (Sigma-Aldrich, St. Louis, USA, CAS: 10034-99-8)	0.150
**GYM medium** (prepared in the lab; composition from Leibniz Institute DSMZ-German Collection of Microorganisms and Cell Cultures GmbH, Germany, https://www.dsmz.de/)	
Glucose (cell-culture grade, PanReac Applichem Barcelona, Spain, CAS: 50-99-7)	4
Yeast Extract (Sigma-Aldrich, St. Louis, USA, CAS: 8013-01-2)	4
Malt Extract (Standard Carl Roth GmbH, Karlsruhe, Germany, CAS:8002-48-0)	10
**Lysogeny medium (LB)** (prepared in the lab; composition from Leibniz Institute DSMZ-German Collection of Microorganisms and Cell Cultures GmbH, Germany, https://www.dsmz.de/)	
Tryptone (Sigma-Aldrich, St. Louis, USA, CAS: 91079-40-2)	10
Yeast Extract (Sigma-Aldrich, St. Louis, USA, CAS: 8013-01-2)	5
NaCl (Sigma-Aldrich, St. Louis, USA, CAS: 7647-14-5)	10
**Nutrient medium (NB)** (procured from HiMedia, Mumbai, India)	
Peptone	15
Yeast Extract	3
NaCl	6
Dextrose	1
**Potato dextrose medium (PDB)** (procured from HiMedia, Mumbai, India)	
Potato infusion	200
Dextrose	20

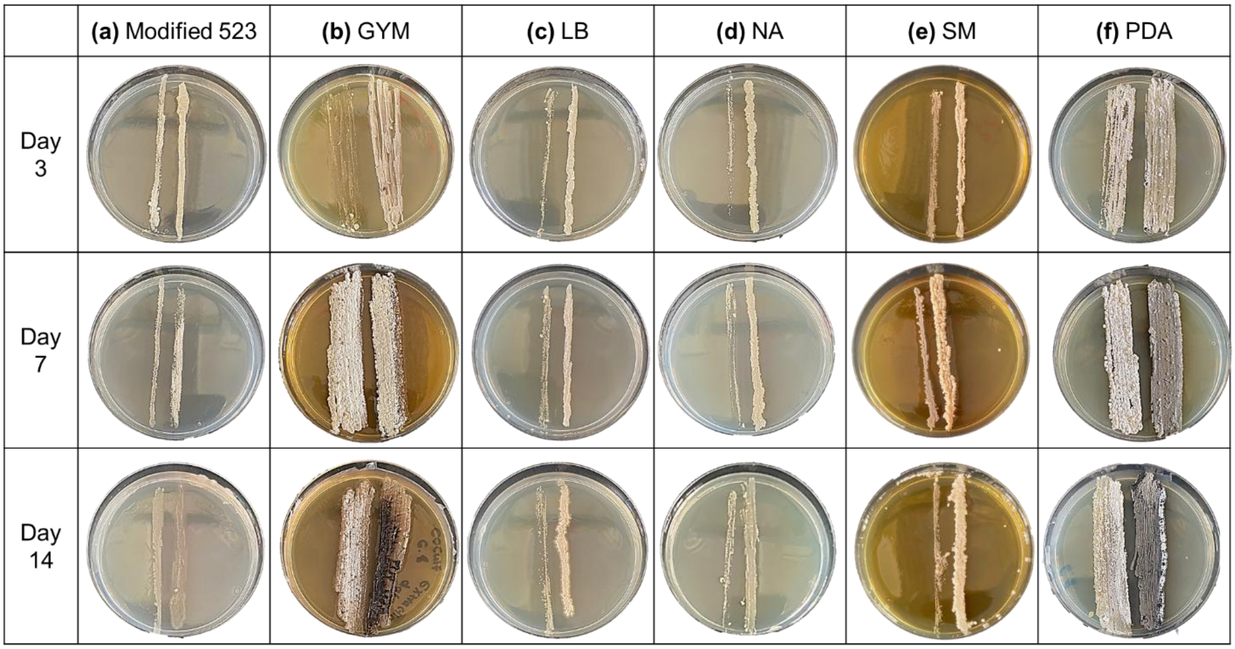

Figure S1. Chemical OSMAC approach-assisted phenotypic characteristics of *S. ciscaucasicus* GS2 (left side of agar plates) co-cultivated with *S. pulveraceus* ES16 (right side of agar plates) on six different agar media for 14 days. **(a)** Modified 523 medium. **(b)** Glucose Yeast Malt medium (GYM). **(c)** Lysogeny medium (LB). **(d)** Nutrient agar (NA). **(e)** Streptomyces medium (SM). **(f)** Potato dextrose agar (PDA).

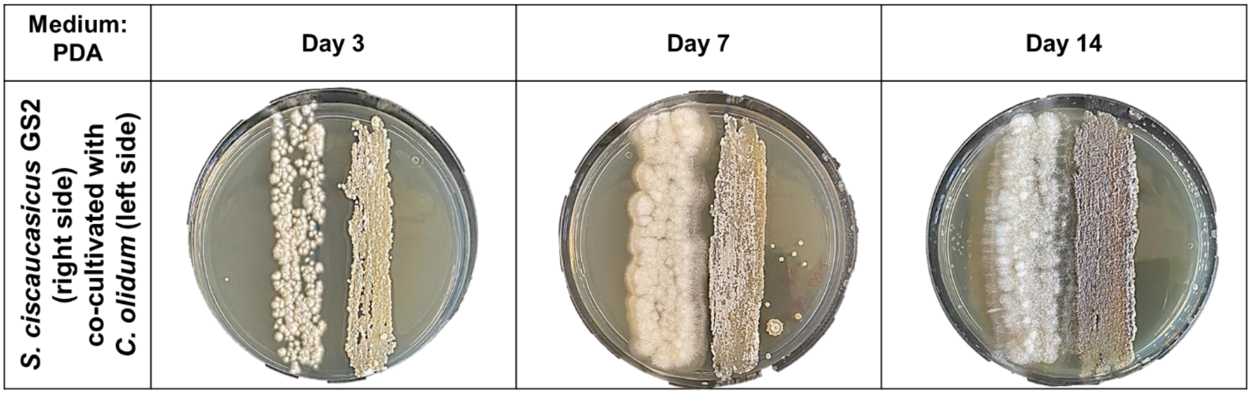

Figure S2. Chemical OSMAC approach-assisted phenotypic characteristics of *S. ciscaucasicus* GS2 (right side of agar plates) co-cultivated with *C. olidum* (left side of agar plates) on potato dextrose agar (PDA) for 14 days.

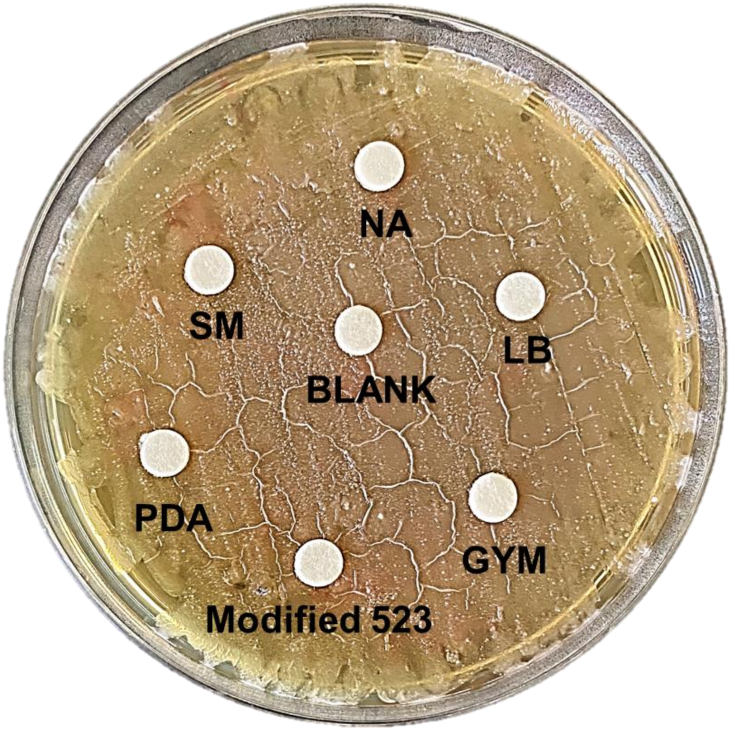

Figure S3. Disc diffusion assay to enumerate the antimicrobial or antagonistic efficacies of extracts of *S. ciscaucasicus* GS2 containing the produced siderophores against *S. pulveraceus* ES16 in six different media *viz*. modified 523 medium, glucose yeast malt medium (GYM), lysogeny medium (LB), nutrient agar (NA), Streptomyces medium (SM), and potato dextrose agar (PDA). A representative SM agar plate is shown. No antimicrobial or antagonistic activity (i.e., no zone of inhibition) was observed.
